# Bibliometric Analysis of 100 Top-Cited Articles in Gastric Disease

**DOI:** 10.1155/2020/2672373

**Published:** 2020-05-15

**Authors:** Fangfang Yuan, Jizhen Cai, Bin Liu, Xiaowei Tang

**Affiliations:** ^1^The 3rd Xiangya Hospital, Central South University, Changsha, Hunan Province 410000, China; ^2^Department of Hematology and Key Laboratory of Non-resolving Inflammation and Tumor, The 3rd Xiangya Hospital, Central South University, Changsha, Hunan Province 410000, China; ^3^Department of Emergency, The First Hospital of Changsha, Changsha, Hunan Province 410000, China; ^4^Department of Gastroenterology, Affiliated Hospital of Southwest Medical University, Luzhou 646099, China

## Abstract

**Objectives:**

The bibliometric analysis uses the citation count of an article to measure its impact in the scientific community, yet there is still no comprehensive summary of gastric disease researches via bibliometric analysis. We aimed to evaluate the situations and trends of the most cited articles in gastric disease via bibliometric analysis and to provide physicians a practical guide in assessing the most influential articles written on this subject.

**Methods:**

The 100 top-cited articles in gastric disease were compiled using Web of Science. The articles selected were evaluated for their number of citations, year of publication, country of origin, type of study, and others.

**Results:**

The database had 484,281 articles published between 1965 and 2019. The most cited article received 4,017 citations and the least received 604, with a mean of 1,149 citations. We classified the articles into seven categories: gastric cancer (*n* = 53), Helicobacter pylori (*n* = 17), ulcer (*n* = 7), gastrointestinal stromal tumors (*n* = 6), gastritis (*n* = 5), gastric bypass surgery (*n* = 2), and others (*n* = 10). Altogether, 69 of the articles were from the USA (*n* = 41), the UK (*n* = 17), and Japan (*n* = 11). Among all the institutions, Royal Perth Hospital led the list with 5 articles. One-quarter of authors owned three or more of these top-cited articles. The 100 papers were published in 33 journals, and most of them were clinical researches (*n* = 47).

**Conclusions:**

Our study provides a historical perspective for the scientific progress of gastric disease, and the articles of significant findings that contributed great impact on the prevention and treatment of gastric disease had been identified.

## 1. Introduction

The excessive medical papers published in journals in Internet databases have led to an overabundance of information, causing it more and more difficult for physicians and researchers to quickly find the body of knowledge of their interest, to which we ought to pay lots of attention. Therefore, there is an imperative need to improve a practitioner' capability to efficiently obtain the approach to the useful articles in his research field.

In the domain of medicine, although the number of citations that one article receives is not necessarily a measure of its academic quality, it could reflect how celebrated that article has been in its branch of learning, and the implication being that the greater the worth of the paper, the more times it would be cited [1]. Although the value of citation times has been debated, a higher number of citations are a direct proxy for a paper's recognition in its scientific field [2]. The establishment of a citation rank list identifies a published work that has the greatest intellectual influence [3]. Top-cited papers in medical journals also serve an important role to educate and encourage the next generation of physicians [4].

Identification of top-cited articles has been conducted in various medical fields which include trauma and orthopaedic surgery [5], plastic surgery [6], general surgery [7], and oncology [8]. Although the researches of gastric disease remain one of the major concerns in the medical and health fields in recent decades, there is a paucity of literature concerning top article citations on gastric disease, yet there is still no comprehensive summary of gastric disease researches in recent decades via bibliometric analysis. Those articles of significant findings and contributed great impact on the prevention and treatment of gastric disease have not been identified and assembled.

Thus, the goal of this study was to identify and analyze the 100 most cited relevant articles published in the peer-reviewed biomedical literature from 1965 to 2017 using the Internet version of the Institute for Scientific Information (ISI) Web of Science database and to find what made them of great value in the medical realm of gastric diseases.

## 2. Methods

**Figure 1:**
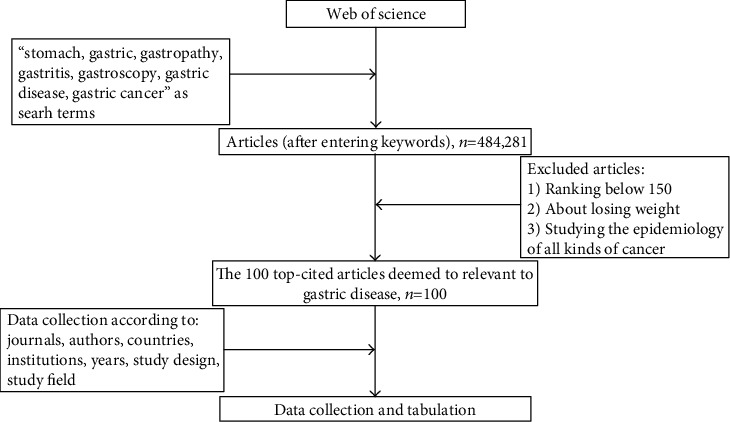
Flow chart showing the methodology used in the study.

To recognize the 100 top-cited articles related to the field of gastric diseases, the authoritative and professional citation indexing database, Thomson Reuters Web of Science, was used by two independent researchers on November 20, 2019 (Web of Knowledge, 2019). To ensure the breadth and relevance of the search scope, the keywords were constantly filtered and finally were established after the number of articles stabilized at 484,281 in Figure 1. And then, “stomach, gastric, gastropathy, gastritis, gastroscopy, gastric disease, gastric cancer” which were included in the title, abstract, or topic were the final set of search terms. In this step, no articles need to be excluded [9].

After confirming the search scope, the next step was to recognize the 100 top-cited articles from the 484,281 articles, which were expressed in a descending numerical order. In order to screen the relevant articles, two independent researchers, respectively, read the abstract or full text of each search result, screened the relevant articles according to the inclusion and exclusion criterion, and manually extracted data concerning the first and second authors, journal name, institution, year of publication, country of origin, total number of citations, the type of study, and the topic. Articles without full text and other complete information could be obtained by linking to other search platforms such as PubMed. About our selection criteria, we excluded (i) articles studying the relationship of endocrine function of stomach and losing weight and (ii) articles studying the epidemiology of all kinds of cancer [10]. All authors contributed to the study conception and design. Data were extracted from each of the articles by Fangfang Yuan, Jizhen Cai, and Bin Liu, and then checked by Xiaowei Tang. All the data were analyzed by Fangfang Yuan and Xiaowei Tang. The first draft of the manuscript was written by Fangfang Yuan and Xiaowei Tang, and all authors commented on previous versions of the manuscript. All the authors read and approved the final manuscript.

## 3. Results

### 3.1. The Basic Characteristics of 100 Top-Cited Articles

The final 100 top-cited articles were listed in Online Resource 1 (Table S1) in a descending order to the number of citations per paper. The article with the largest citation number, 4017 times, was about a histoclinical classification of gastric cancer published in 1965 [11]. The article with the least citations (604 times) was about atrophic gastritis and Helicobacter pylori (Hp) infection in patients with reflux esophagitis treated with omeprazole or fundoplication published in 1996 [12].

**Figure 2:**
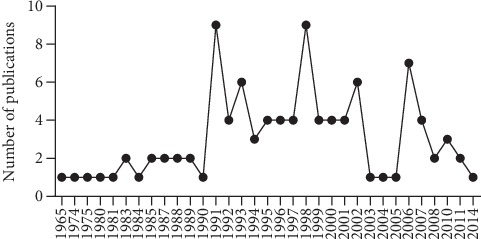
Distribution of top-cited articles per year.

These articles were published between 1965 and 2014. It could be seen from the timeline diagram of Figure 2 that there were two peaks in the region of 1990-2003 and 2005-2014, respectively. And we demonstrated that most (84%) of the highly cited articles in gastric disease were published in the period from 1990 to 2014.

### 3.2. Journals and Authors

Journals of the top 100-cited articles were listed in Table 1 in a descending order by the number of articles with the average number of citations per paper and the impact factor (2016-2017). There were altogether 33 journals included: *Lancet* (*n* = 18), *New England Journal of Medicine* (*n* = 17), *Gastroenterology* (*n* = 8), *Cancer Research* (*n* = 5), *Nature* (*n* = 5), *Science* (*n* = 5), *Gut* (*n* = 4), *American Journal of Surgical Pathology* (*n* = 3), and others (*n* = 35).

There were 23 authors who contributed more than 2 top-cited articles listed as first or second author. This list is led by Marshall BJ, who authored 5 of the T100 articles. These 23 authors are listed in Table 2.

### 3.3. Countries and Institutions

Countries and institutions of origin of the T100 articles were, respectively, listed in Tables 3 and 4. Concerning the countries of origin, a total of 14 different countries contributed these 100 articles. The USA (*n* = 41) with nearly half of the articles, became the most prolific country, followed by the UK (*n* = 17), Japan (*n* = 11), Germany (*n* = 9), Netherlands (*n* = 6), Australia (*n* = 4), Belgium (*n* = 3), and others.

However, the institution with the largest number of papers was led by the Royal Perth Hospital of the UK (*n* = 5). Four institutions produced three articles: Louisiana State University Medical Center, New Orleans, Louisiana of the USA; Stanford University School of Medicine, California of the USA; Vanderbilt University School of Medicine, Nashville, Tennessee of the USA, and University Hospital Gasthuisberg of Belgium. The remaining 13 different institutions produced two articles, respectively.

### 3.4. Study Design and Study Field of the Top-Cited Articles

**Figure 3:**
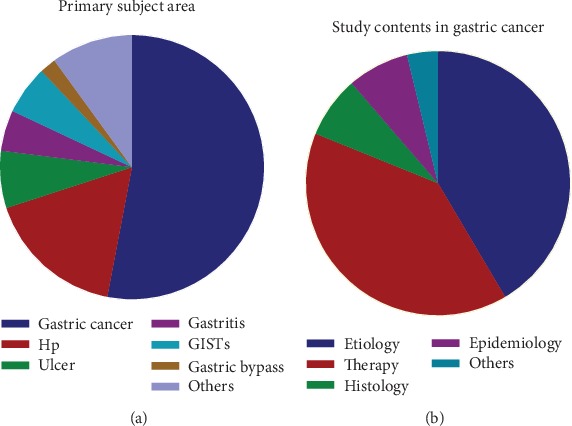
(a) Primary subject matter of the highly cited articles by percentage. (b) Distribution of study contents in gastric cancer divided by percentage (Hp: Helicobacter pylori; GISTs: gastrointestinal stromal tumors).

**Figure 4:**
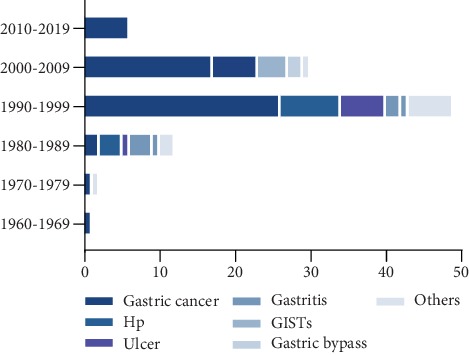
Distribution of study subjects in T100 divided by decade.

Among the primary study field of the T100 articles on gastric diseases, the topics related to cancer occupied the most articles (*n* = 53). Researches related to Hp accounted for the second (*n* = 17) followed by ulcer (*n* = 7), gastrointestinal stromal tumors (GISTs) (*n* = 6), gastritis (*n* = 5), gastric bypass (*n* = 2), and others (*n* = 10) (Figure 3(a)). And we also showed the distribution of T100 study subjects by decade of publication in Figure 4.

The study contents of T100 articles could be categorized into etiology, therapy, histology, epidemiology, and others. Etiology took the largest proportion of the T100 articles (*n* = 22), followed by therapy (*n* = 21) (Figure 3(b)).

**Figure 5:**
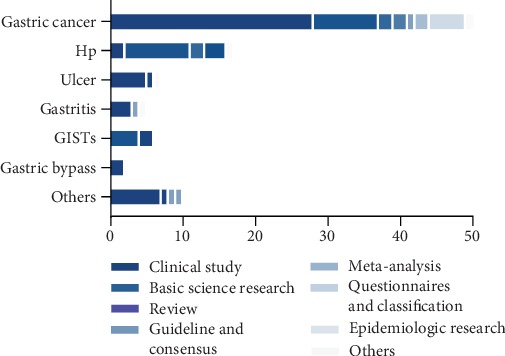
Distribution of article type in T100 divided by study contents.

For the study designs of the 100 top-cited articles, clinical study accounted for 47% including gastric cancer (*n* = 28), Hp (*n* = 2), ulcer (*n* = 5), gastritis (*n* = 3), GISTs (*n* = 0), gastric bypass (*n* = 2), and others (*n* = 7), followed by basic science research 23%, review 7%, guideline and consensus 5%, questionnaires and clarification 5%, epidemiologic research 5%, meta-analysis 2%, and others 6% (Figure 5).

**Figure 6:**
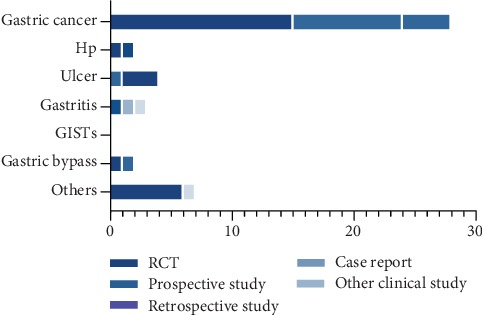
Distribution of article type in clinical study divided by study contents.

The total 47 clinical study includes randomized controlled trials (RCTs) (*n* = 27), prospective study (*n* = 11), retrospective study (*n* = 6), case report (*n* = 1), and others (*n* = 2). And most of the studies that focus on gastric cancer are RCT (*n* = 15), followed by prospective study (*n* = 9) (Figure 6).

## 4. Discussion

It cannot be doubted that, within any given field, citation analysis can always provide enormous information with journals, institutions, and authors, which is available for identifying landmark papers and high-impact journals. It not only provides a historical prospect on scientific headway in the field of gastric diseases but also reveals the trend of gastric disease investigation [13]. The bibliometric analysis can also help to recognize major advances in biomedical research, characterize the specific subject, and provide insight on the most popular themes in gastric disease in the past always.

The 100 top-cited articles published in journals from 1965 to 2014 were cited 604 to 4017 times. The list of the articles identified topics that reflect changing trends in gastric disease research over the past 49 years. Although it may be impossible to carry out a detailed analysis of all the 100 highly cited articles, some observations could be found. Our current existing study summarizes the features of influential articles in gastric diseases during the past 49 years.

We found that 41 of the 100 articles in the top 100 list are from the USA, which confirms the tremendous impact on medical science research in the USA as its large scientific population and the sufficient financial resources which are available to the scientific communities. The country's dominance was also found in other clinical disciplines, including Psychiatry [14], Urology [15], Respiratory [16], General Surgery [7], Cardiology [17], and Critical Care Medicine [18]. It is generally believed that the reciprocity between the research yield and GDP should be an important issue in biometric analysis. With no surprise, American researchers would usually prefer local articles in their citation process, and the American reviewers possibly prefer US papers [19, 20].

The top-cited papers were not equally allocated across all gastric disease subspecialties. Considering the severity of stomach diseases in different categories, it is not surprising that among all the most cited articles, articles related to gastric cancer figured remarkably in the top 100 list, and 49 in this group are more likely to rank highest. With the continuous increase of cancer worldwide, which leads to an increasing number of mortality every year, the treatment of gastric cancer has been well studied and specifically counted for 19 papers.

In addition, articles about Hp, which plays a vital role in the pathology of gastritis and gastric cancer [21], account for 16% of the list. Among all five articles of Marshall BJ, it could be founded that the principal study direction was Hp, which is one of the most important microbes in this subject. Marshall BJ and his team expounded the initial discovery of Hp and the relationship between Hp infection and gastritis or peptic ulcer disease. These findings form an important basis for future researches and reflect on the direction of researches in the next 40 years. Afterwards, there were more clear understandings about the pathogen, diagnosis, treatment, and prognosis of gastritis and peptic ulcer disease.

In general, among various kinds of study designs, RCTs are considered to provide the highest quality evidence [22]. Through further scrutiny, it can be observed that of the 39 articles citing more than 1000 citations, respectively, 19 are clinical researches, which are followed by basic science researches (9 articles) and RCTs (8 articles), prospective studies (7 articles), and retrospective studies (2 articles).

Influential publications are more likely to be cited by the scientific community. These citations form the basis of the impact factor, which quantifies the average citations of the articles published within the journal during a specific period [14]. While the 100 top-cited articles appeared in a total of 32 journals, *Lancet* or the *New England Journal of Medicine* was the sources for over one-third of the articles—both the journals have an impact factor > 40 (2017-2018). Our findings may support the application of a bibliometric concept suggested by Brookes' Bradford's law, which holds the point of view that most researchers get their citations from a few main journals in their respective field of expertise. However, once the researchers deviate from these core journals, their citation frequency and impact are weakened. Thus, this tendency leads to a large percentage of citations which stem from a few major journals.

To the best of our knowledge, this is the first bibliometric analysis to reveal the top citations in the field of gastric disease. It has been reported that time of issuing can have much effect on the citation ranking of an article, because an article's citations logically depend on its publication time, as citations would accumulate over time [16].

The articles were published between 1965 and 2014; we demonstrated that most (77 articles) of the top-cited articles in gastric diseases were in the period from 1991 to 2007. This is analogous to most other bibliometric analyses, which generally found that the true impact of an article often cannot be accurately precisely assessed for at least 2 decades after it was published [23–27]. On the contrary, as time passes, even classic papers may gradually be less cited because their findings have been absorbed into current knowledge without further need for reference [28]. Absence of any article before 1965 in the top-cited list might indicate the impermanent usefulness of outmoded articles in the modern period of time. Moreover, limitations in databases for retrieving older articles and the lack of online and internet resources in the pre-1990s might also have contributed to this descending trend of citation [17].

It should be kept in mind that there were some weaknesses in our research. First, the main weakness was due to our methodology that was straightforward and duplicable. However, restricting articles to those that explicitly refer to gastric diseases in the title could result in potential omissions of some relevant articles. Second, the use of citations contained problems such as the lack of correction for self-citations, the lack of counting possible citations in book chapters and preference of peers to cite review articles or articles from the journal in which they seek to publish their own work. Third, the inherent problems associated with citation analyses, such as the bias linked to relying on the total number of times an article is cited, must be noted. This could preferentially favor older articles [28]. Furthermore, there were also other potential influences that may affect the citation rates. For instance, we only used a single electronic medical database to conduct our research. Therefore, we should keep in mind the significant differences between the various databases; it is possible that our list would become kind of different if we used Google scholar or any other databases instead of Web of Science [29].

## 5. Conclusion

In conclusion, our bibliometric analysis of the top-cited papers allows the recognition of major advances and progress in the area of gastric disease, which can help identify the significant researches of gastric disease, accelerate the progress of study, and reveal the trends of investigation.

## Figures and Tables

**Table 1:** Journals in which the top 100 cited gastric disease articles were published.

Rank	Journals	No. of articles (%)	Average no. of citations per paper	Impact factor^†^ (2018)
1	Lancet	18	1124.778	59.102
2	New England Journal of Medicine	17	1405.059	70.67
3	Gastroenterology	8	711.125	19.809
4a	Cancer research	5	1134.8	8.378
4b	Nature	5	1728.6	43.07
4c	Science	5	1237	41.063
5	Gut	4	1045	17.943
6a	American Journal of Surgical Pathology	3	1613.333	6.155
6b	Annals of Internal Medicine	3	848	19.315
7a	Journal of American medical association	2	1380	51.273
7b	Annals of Surgery	2	825	9.476
7c	Cancers	2	1105.5	6.162
7d	Clinical Microbiology Reviews	2	948.5	17.75
7e	Gastric Cancer	2	790	5.554
7f	Journal of Clinical Oncology	2	899	28.245
7 g	Lancet Oncology	21	852	35.386
7 h	Medical Journal of Australia	2	833	5.332
8a	Alimentary Pharmacology & Therapeutics	1	1033	7.731
8b	American Journal of Gastroenterology	1	706	10.241
8c	Archives of Pathology & Laboratory Medicine	1	773	4.151
8d	BMJ (Clinical Research Ed.)	1	1150	27.604
8e	British Journal of Cancer	1	921	5.416
8f	Cancer Cell	1	645	23.916
8 g	Cell	1	859	36.216
8 h	Journal of Gastroenterology and Hepatology	1	869	3.632
8i	Journal of Pediatric Surgery	1	1328	2.092
8j	Nature Reviews Cancer	1	1094	51.848
8 k	Surgical Laparoscopy & Endoscopy	1	840	1.345
8 l	American Journal of Gastroenterology	1	774	10.241
8 m	Virchows Archiv	1	1107	2.868
8n	World Journal of Gastroenterology	1	1036	3.411
8o	World Journal of Surgery	1	607	2.768

^†^Journal impact factor based on Thomson Reuters Web of Knowledge Journal Citation Reports Ranking (2018).

**Table 2:** Authors with two or more top-cited articles.

Rank	Author	No. of articles	First	Second
1	Marshall BJ	5	4	1
2a	Correa P	3	3	
2b	Miettinen M	3	3	
2c	Parsonnet J	3	3	
2d	Van Cutsem E	3	1	2
3a	Bang YJ	2	2	
3b	Bayerdorffer E	2	2	
3c	Blaser MJ	2	1	1
3d	Blot MJ	2	1	1
3e	Bonenkamp JJ	2	2	
3f	Cohen H	2		2
3 g	Cuschieri A	2	2	
3 h	El-Omar EM	2	2	
3i	Friedman GD	2		2
3j	Graham DY	2	2	
3 k	Japanese Gastric Canc. Assco.	2	2	
3 l	Kuipers EJ	2	2	
3 m	Malfertheiner P	2	2	
3n	Megraud F	2		2
3o	Neubauer A	2		2
3p	Songun I	2	1	1
3q	Warren JR	2	1	1
3r	Wong BCY	2	1	1

**Table 3:** Countries of origin of the top-cited articles.

Rank	Countries	No. of articles
1	USA	41
2	UK	17
3	Japan	11
4	Germany	9
5	The Netherlands	6
6	Australia	4
7	Belgium	3
8a	Italy	2
8b	South Korea	2
9a	Canada	1
9b	Hong Kong, China	1
9c	Denmark	1
9d	Finland	1
9e	New Zealand	1

USA: United States of America; UK: United Kingdom.

**Table 4:** Institutions of origin with two or more top-cited articles.

Rank	Institutions	No. of articles
1	Royal Perth Hospital, Western Australia, UK	5
2	Louisiana State University Medical Center, New Orleans, Louisiana, USA	3
3a	Stanford University School of Medicine, California, USA	3
3b	Vanderbilt University School of Medicine, Nashville, Tennessee, USA	3
3c	University Hospital Gasthuisberg, Belgium	3
4a	Department of Soft Tissue Pathology, Armed Forces Institute of Pathology, Washington, USA	2
4b	Kitasato Univ., Sch. Med., Dept. Internal Med., Sagamihara, Kanagawa 228, Japan	2
4c	Kyushu University, Fukuoka, Japan	2
4d	Leiden Univ. Med Ctr., Leiden, Netherlands	2
4e	Mayo Clinic and Foundation, Rochester, Minnesota, USA	2
4f	National Cancer Institute, NIH, Bethesda, Maryland, USA	2
4 g	National Cancer Center Hospital, Tokyo, Japan	2
4 h	Osaka University Medical School, Yamadaoka, Suita, Japan	2
4i	The Free University Hospital, Amsterdam, Netherlands	2
4j	UCL Medical School, London, UK	2
4 k	University of Munich, Germany	2
4 l	Veterans Affairs Medical Center, Dallas, Texas, USA	2
4 m	Washington University School of Medicine, Jewish Hospital of St. Louis, and Division of Neuropathology, St. Louis, Missouri, USA	2

## Data Availability

The data used to support the findings of this study are available from the corresponding author upon request.

## Supplementary Materials

Supplementary Materials Table S1: the 100 top-cited papers in gastric disease.
