# Supplementary material for: Bibliometric Analysis of 100 Top-Cited Articles in Gastric Disease
Source: Biomed Res Int. 2020 May 15;2020:2672373. doi: 10.1155/2020/2672373 (PMC7245662; doi:10.1155/2020/2672373)
Supplement: Supplementary Materials — Table S1: the 100 top-cited papers in gastric disease. [file 2672373.f1.pdf]

Bibliometric analysis of 100 top-cited articles in gastric disease

Fangfang Yuan[1,2], Jizhen Cai[2] , Bin Liu[3], Xiaowei Tang[4,5]

Table S1 The 100 top-cited papers in gastric disease.

Rank	Title	No. of citations	Citation per year
1	The two histological main types of gastric carcinoma: diffuse and so-called intestinal-type carcinoma. A attempt at a histo-clinical classification. Acta Pathol Microbiol Scand. 1965;64:31-49.	4017	77.25
2	Unidentified curved bacilli in the stomach of patients with gastritis and peptic ulceration. Lancet. 1984;1(8390):1311-5.	3474	105.27
3	The complete genome sequence of the gastric pathogen Helicobacter pylori. Nature. 1997;388(6642):539-47.	3330	166.5
4	Classification and grading of gastritis. The updated Sydney System. International Workshop on the Histopathology of Gastritis, Houston 1994. Am J Surg Pathol. 1996;20(10):1161-81.	3304	157.33
5	Helicobacter pylori infection and the risk of gastric carcinoma. N Engl J Med. 1991;325:1127-31.	3113	119.73
6	Gain-of-function mutations of c-kit in human gastrointestinal stromal tumors. Science. 1998;279:577-80.	2787	146.68
7	Helicobacter pylori infection and the development of gastric cancer. N Engl J Med. 2001;345:784-9.	2490	155.63
8	Unidentified curved bacilli on gastric epithelium in active chronic gastritis. Lancet. 1983;1:1273-5.	2432	71.53
9	Perioperative chemotherapy versus surgery alone for resectable gastroesophageal cancer. N Engl J Med. 2006 Jul 6;355(1):11-20.	2405	218.64
10	Chemoradiotherapy after surgery compared with surgery alone for adenocarcinoma of the stomach or gastroesophageal junction. N Engl J Med. 2001;345:725-30.	2048	128
11	Human gastric carcinogenesis: a multistep and multifactorial process--First American CancerSociety Award Lecture on Cancer Epidemiology and Prevention. Cancer research. 1992;52:6735-40.	2023	80.92
12	Genomic-sequence comparison of two unrelated isolates of the human gastric pathogen Helicobacter pylori. Nature. 1999;397:176-80.	1988	110.4
13	Rising incidence of adenocarcinoma of the esophagus and gastric cardia. JAMA. 1991;265:1287-9.	1923	73.96
14	Interleukin-1 polymorphisms associated with increased risk of gastric cancer. Nature. 2000;404:398-402.	1679	98.76
15	Regression of primary low-grade B-cell gastric lymphoma of mucosa-associated lymphoid tissue type after eradication of	1666	69.42

	Helicobacter pylori. Lancet. 1993;342(8871):575-7.		
16	Changing patterns in the incidence of esophageal and gastric carcinoma in the United States. Cancer. 1998;83:2049-53.	1568	82.53
17	Helicobacter pylori infection. N Engl J Med. 2002;347:1175-86.	1564	104.27
18	Helicobacter pylori infection and gastric carcinoma among Japanese Americans in Hawaii. N Engl J Med. 1991;325:1132-6.	1556	59.85
19	Plasma ghrelin levels after diet-induced weight loss or gastric bypass surgery. N Engl J Med. 2002;346:1623-30.	1479	98.6
20	Helicobacter pylori infection and gastric lymphoma. N Engl J Med. 1994;330:1267-71.	1466	63.74
21	Helicobacter pylori-associated gastritis and primary B-cell gastric lymphoma. Lancet. 1991;338:1175-6.	1441	55.42
22	Trastuzumab in combination with chemotherapy versus chemotherapy alone for treatment of HER2-positive advanced gastric or gastro-oesophageal junction cancer (ToGA): a phase 3, open-label, randomised controlled trial. Lancet. 2010;376: 687-697.	1440	205.71
23	Current concepts in the management of Helicobacter pylori infection: the maastricht III consensus report. Gut. 2007;56(6):772-81.	1337	133.7
24	Gastrostomy without laparotomy: a percutaneous endoscopic technique. J Pediatr Surg. 1980;15(6):872-5.	1328	35.89
25	Long-term mortality after gastric bypass surgery. N Engl J Med. 2007;357:753-761.	1317	131.7
26	Adjuvant chemotherapy for gastric cancer with S-1, an oral fluoropyrimidine. N Engl J Med. 2007;357:1810-1820.	1245	124.5
27	Infection with Helicobacter pylori strains possessing cagA is associated with an increased risk of developing adenocarcinoma of the stomach. Cancer Res. 1995;55(10):2111-5.	1228	55.82
28	The Vienna classification of gastrointestinal epithelial neoplasia. Gut. 2000;47:251-5.	1177	69.24
29	Association between infection with Helicobacter pylori and risk of gastric cancer: evidence from a prospective investigation. BMJ. 1991;302: 1302-1305.	1150	44.23
30	Gastrointestinal stromal tumors--definition, clinical, histological, immunohistochemical, and molecular genetic features and differential diagnosis. Virchows Arch. 2001;438(1):1-12.	1107	69.19
31	Trastuzumab in combination with chemotherapy versus chemotherapy alone for treatment of HER2-positive advanced gastric or gastro-oesophageal junction cancer (TOGA): a phase 3, open-label, randomised controlled trial (vol 376, pg 687, 2010). Lancet. 2010;376: 1302-1302.	1098	156.86
32	Extended lymph-node dissection for gastric cancer. N Engl J Med. 1999;340:908-14.	1095	60.83

33	Helicobacter pylori and gastrointestinal tract adenocarcinomas. Nat Rev Cancer. 2002;2(1):28-37.	1094	72.93
34	Phase III study of docetaxel and cisplatin plus fluorouracil compared with cisplatin and fluorouracil as first-line therapy for advanced gastric cancer: a report of the V325 Study Group. J Clin Oncol. 2006;24(31):4991-7.	1051	95.55
35	Attempt to fulfil Koch's postulates for pyloric Campylobacter. Med J Aust. 1985;142(8):436-9.	1046	32.69
36	Epidemiology of gastric cancer. World journal of gastroenterology. 2006;12:354-62.	1036	94.18
37	Current concepts in the management of Helicobacter pylori infection--the Maastricht 2-2000 Consensus Report. Aliment Pharmacol Ther. 2002;16(2):167-80.	1033	68.87
38	Helicobacter pylori. Clinical microbiology reviews. 1997;10:720-41.	1020	51
39	E-cadherin germline mutations in familial gastric cancer. Nature. 1998;392:402-5.	1010	53.16
40	A human model of gastric carcinogenesis. Cancer research. 1988;48:3554-60.	995	34.31
41	S-1 plus cisplatin versus S-1 alone for first-line treatment of advanced gastric cancer (SPIRITS trial): a phase III trial. Lancet Oncol. 2008;9(3):215-21.	992	110.22
42	Endoscopic mucosal resection for treatment of early gastric cancer. Gut. 2001;48:225-9.	989	61.81
43	Risk for serious gastrointestinal complications related to use of nonsteroidal anti-inflammatory drugs. A meta-analysis. Ann Intern Med. 1991;115(10):787-96.	969	37.27
44	Japanese classification of gastric carcinoma: 3rd English edition. Gastric Cancer. 2011;14(2):101-12.	957	159.5
45	Effect of treatment of Helicobacter pylori infection on the long-term recurrence of gastric or duodenal ulcer. A randomized, controlled study. Annals of internal medicine. 1992;116:705-8.	955	38.2
46	Prospective double-blind trial of duodenal ulcer relapse after eradication of Campylobacter pylori. Lancet. 1998;2:1437-42.	949	49.95
47	Patient survival after D1 and D2 resections for gastric cancer: long-term results of the MRC randomized surgical trial. Surgical Co-operative Group. Br J Cancer. 1999;79(9-10):1522-30.	921	51.17
48	Gastric cancer originating from bone marrow-derived cells. Science. 2004 ;306(5701):1568-71.	879	67.62
49	Pathogenesis of Helicobacter pylori infection. Clinical microbiology reviews. 2006;19:449-90.	877	79.73
50	Campylobacter pyloridis-associated chronic active antral gastritis. A prospective study of its prevalence and the effects of antibacterial and antiulcer treatment. Gastroenterology. 1988;94(1):33-40.	877	30.24
51	Translocation of Helicobacter pylori CagA into gastric epithelial cells	871	51.24

	by type IV secretion. Science. 2000;287:1497-500.		
52	The Sydney System: histological division. Journal of gastroenterology and hepatology. 1991;6:209-22	869	33.42
53	Causal relationship between the loss of RUNX3 expression and gastric cancer. Cell. 2002;109:113-24.	859	57.27
54	Laparoscopy-assisted Billroth I gastrectomy. Surgical laparoscopy & endoscopy. 1994;4:146-8.	840	36.52
55	Helicobacter pylori eradication to prevent gastric cancer in a high-risk region of China: a randomized controlled trial. JAMA. 2002;291:187-94.	837	55.8
56	Outcomes after laparoscopic Roux-en-Y gastric bypass for morbid obesity. Ann Surg. 2000;232(4):515-29.	830	48.82
57	Attachment of Helicobacter pylori to human gastric epithelium mediated by blood group antigens. Science. 1993;262:1892-5.	827	34.46
58	Helicobacter pylori virulence and genetic geography. Science. 1999;284:1328-33.	821	45.61
59	Relevant prognostic factors in gastric cancer: ten-year results of the German Gastric CancerStudy. Ann Surg. 1998;228:449-61.	820	43.16
60	High blood alcohol levels in women. The role of decreased gastric alcohol dehydrogenase activity and first-pass metabolism. N Engl J Med. 1990;322:95-9.	811	30.04
61	Gastrointestinal stromal tumors of the stomach: a clinicopathologic, immunohistochemical, and molecular genetic study of 1765 cases with long-term follow-up. Am J Surg Pathol. 2005;29(1):52-68.	785	65.42
62	Regression of primary gastric lymphoma of mucosa-associated lymphoid-tissue type after cure of Helicobacter-pylori infection. Lancet. 1995;345:1591-4.	783	35.59
63	Gastrointestinal stromal tumors: pathology and prognosis at different sites. Semin Diagn Pathol. 2006;23(2):70-83.	782	71.09
64	Helicobacter pylori infection induces gastric cancer in mongolian gerbils. Gastroenterology. 1998;115(3):642-8.	774	40.74
65	Ingestion of Campylobacter pyloridis causes gastritis and raised fasting gastric pH. Am J Gastroenterol. 1987;82(3):192-9.	774	25.8
66	Gastrointestinal stromal tumors: review on morphology, molecular pathology, prognosis, and differential diagnosis. Arch Pathol Lab Med. 2006;130(10):1466-78.	773	70.27
67	Randomised comparison of morbidity after D1 and D2 dissection for gastric cancer in 996 Dutch patients. Lancet. 1995;345(8952):745-8.	761	34.59
68	An international association between Helicobacter pylori infection and gastric cancer. The EUROGAST Study Group. Lancet. 1993;341(8857):1359-62.	753	31.38
69	Gastric stromal tumors. Reappraisal of histogenesis. Am J Surg Pathol. 1983;7(6):507-19.	751	22.09

70	Chemotherapy in advanced gastric cancer: a systematic review and meta-analysis based on aggregate data. J Clin Oncol. 2006;24(18):2903-9.	747	67.91
71	Expression of cyclooxygenase-2 in human gastric carcinoma. Cancer research. 1997;57:1276-80.	743	37.15
72	Campylobacter pylori and peptic ulcer disease. Gastroenterology. 1989;96 :615-25.	731	26.11
73	Effect of ranitidine and amoxicillin plus metronidazole on the eradication of Helicobacter pylori and the recurrence of duodenal ulcer. N Engl J Med. 1993;328:308-12.	724	30.17
74	Inhibition of gastrin and gastric-acid secretion by growth-hormone release-inhibiting hormone. Lancet. 1974;2:1106-9.	713	16.58
75	Surgical treatment of gastric cancer: 15-year follow-up results of the randomised nationwide Dutch D1D2 trial. Lancet Oncol. 2010;11(5):439-49.	712	101.71
76	Prevalence of Helicobacter pylori infection and histologic gastritis in asymptomatic persons. N Engl J Med. 1989;321:1562-6.	709	25.32
77	A model for gastric cancer epidemiology. Lancet. 1975;2:58-60.	709	16.88
78	American college of gastroenterology guideline on the management of Helicobacter pylori infection. Am J Gastroenterol. 2007;102(8):1808-25. Epub 2007 Jun 29.	706	70.6
79	Role of impaired gastric accommodation to a meal in functional dyspepsia. Gastroenterology. 1998;11:1346-52.	705	37.11
80	The response of cells from low-grade B-cell gastric lymphomas of mucosa-associated lymphoid tissue to Helicobacter pylori. Lancet. 1993;342:571-4.	705	29.38
81	E-cadherin gene mutations provide clues to diffuse type gastric carcinomas. Cancer Res. 1994;54:3845-52.	685	29.78
82	Risk for gastric cancer in people with CagA positive or CagA negative Helicobacter pylori infection. Gut. 1997;40:297-301.	677	33.85
83	Postoperative morbidity and mortality after D1 and D2 resections for gastric cancer: preliminary results of the MRC randomised controlled surgical trial. The Surgical Cooperative Group. Lancet. 1996;347:995-9.	677	32.24
84	Measurement of gastric emptying rate of solids by means of a carbon-labeled octanoic acid breath test. Gastroenterology. 1993;104:1640-7.	670	27.92
85	Dyspepsia and dyspepsia subgroups: a population-based study. Gastroenterology. 1992;102:1259-68.	654	26.16
86	E2F1-regulated microRNAs impair TGF beta-dependent cell-cycle arrest and apoptosis in gastric cancer. Cancer Cell. 2008;13(3):272-86.	645	71.67
87	Prognostic value of vascular endothelial growth factor expression in gastric carcinoma. Cancer. 1996;77:858-63.	643	30.62

88	Omeprazole compared with misoprostol for ulcers associated with nonsteroidal antiinflammatory drugs. Omeprazole versus Misoprostol for NSAID-induced Ulcer Management (OMNIUM) Study Group. N Engl J Med. 1998;338(11):727-34.	641	33.74
89	Increased risk of noncardia gastric cancer associated with proinflammatory cytokine gene polymorphisms. Gastroenterology. 2003;124:1193-201.	640	45.71
90	Meta-analysis of the relationship between Helicobacter pylori seropositivity and gastriccancer. Gastroenterology. 1998;114:1169-79.	638	33.58
91	Substituted benzimidazoles inhibit gastric acid secretion by blocking (H+ + K+)ATPase. Nature. 1981;290:159-61.	636	17.67
92	Ramucirumab monotherapy for previously treated advanced gastric or gastro-oesophageal junction adenocarcinoma (REGARD): an international, randomised, multicentre, placebo-controlled, phase 3 trial. Lancet. 2014; 383: 31-39.	630	210
93	Japanese gastric cancer treatment guidelines 2010 (ver. 3). Gastric Cancer. 2011;14(2):113-23.	623	103.83
94	Gastric intramucosal pH as a therapeutic index of tissue oxygenation in critically ill patients. Lancet. 1992;339:195-9.	623	24.92
95	Nonsteroidal anti-inflammatory drug use and increased risk for peptic ulcer disease in elderly persons. Ann Intern Med. 1991;114(4):257-63.	620	23.85
96	Pyloric Campylobacter infection and gastroduodenal disease. Med J Aust. 1985;142(8):439-44.	620	19.38
97	Helicobacter pylori and peptic ulcer disease. N Engl J Med. 1991;324:1043-8.	619	23.81
98	Long-term sequelae of Helicobacter pylori gastritis. Lancet. 1995;345:1525-8.	610	27.73
99	Progress in gastric cancer surgery in Japan and its limits of radicality. World J Surg. 1987;11(4):418-25.	607	20.23
100	Atrophic gastritis and Helicobacter pylori infection in patients with reflux esophagitis treated with omeprazole or fundoplication. N Engl J Med. 1996;334:1018-22.	604	28.76
